# Solid-State Fermentation of Riceberry Rice with Mushroom Mycelium for Enhanced Beta-Glucan Production and Health Applications

**DOI:** 10.3390/molecules30193879

**Published:** 2025-09-25

**Authors:** Jutamat Nacha, Hongyu Chen, Amorn Owatworakit, Kittirat Saharat, Anupong Makeudom, Sunita Chamyuang

**Affiliations:** 1 School of Science, Mae Fah Luang University, Tha-Sut, Muang, Chiang Rai 57100, Thailand; 6551105002@lamduan.mfu.ac.th (J.N.); amorn@mfu.ac.th (A.O.); kittirat.sah@mfu.ac.th (K.S.); 2 Microbial Products and Innovation Research Group, Mae Fah Luang University, Tha-Sut, Muang, Chiang Rai 57100, Thailand; 3 Institute of Edible Fungi, Shanghai Academy of Agricultural Sciences, No. 1000 Jinqi Road, Fengxian District, Shanghai 201403, China; chenhongyu20128@163.com; 4 School of Dentistry, Mae Fah Luang University, Tha-Sut, Muang, Chiang Rai 57100, Thailand; anupong.mak@mfu.ac.th

**Keywords:** anti-cancer, beta-glucan, mushroom mycelium, *P. ostreatus*, prebiotic, Riceberry rice, solid-state fermentation

## Abstract

Beta-glucans (β-glucans), polysaccharides found in cereals and fungi, are recognized for their prebiotic and potential anti-cancer activities, particularly in the colorectal area. This study aims to optimize the production of β-glucan through the solid-state fermentation of germinated Riceberry rice with *Pleurotus ostreatus* and evaluate the bioactivities of the resulting extract. The crude β-glucan extract, obtained with a recovery rate of 54.95% and 79.98% purity, demonstrated an effective extraction process, as confirmed by thermogravimetric analysis (TGA). Fourier-transform infrared spectroscopy (FTIR) analysis verified the presence of β-1,3/1,6-glycosidic linkages, characteristic of the bioactive β-glucans found in yeast and mushrooms. The biological assessment demonstrated the extract’s functional properties. At a concentration of 1 mg/mL, the crude β-glucan extract significantly promoted the growth of probiotics *Lacticaseibacillus rhamnosus* and *Bacillus coagulans*, exhibiting high Prebiotic Index (PI) values of 6.36 ± 0.72 and 115.70 ± 10.19, respectively, with PI values indicating strong prebiotic potential. For comparison, the standard prebiotic inulin yielded PI values of 0.41 ± 0.09 and 90.53 ± 2.28 for the same respective bacteria, highlighting the superior performance of the fungal-fermented β-glucan. Furthermore, the extract displayed efficacy in inhibiting colon cancer cells in preliminary in vitro tests. It reduced the viability of the SW480 colorectal cancer cell line by 66.23% and induced cell death in 27.94 ± 0.93% of the cells after 48 h of treatment, performing comparably to a commercial yeast β-glucan standard. Crucially, the extract showed no significant cytotoxicity toward the normal human colon cell line, CCD-841 CoN. These findings highlight the promising method of fungal solid-state fermentation on germinated Riceberry rice in the production of high-purity, bioactive β-glucans for use in functional foods.

## 1. Introduction

The increasing global demand for functional foods has stimulated research into bioactive compounds that confer specific health benefits. Among these, β-glucans, a class of polysaccharides found in the cell walls of cereals, fungi, and bacteria, have emerged as prominent candidates due to their diverse physiological functions [1]. Structurally, β-glucans consist of β-D-glucose monomers linked by (1,3), (1,4), or (1,6) glycosidic bonds. These polysaccharides have been credited with a range of health benefits, including immunomodulation, prebiotic effects that promote gut health, reduce blood cholesterol, and offer potential anti-colorectal cancer activity. Regarding the effect of anti-cancer, beta-glucan (β-glucan) from *P. ostreatus* stimulates the immune system, particularly the functions of macrophages, T-cells, and NK cells, enabling the inhibition of cancer cell growth and inducing apoptosis in cancer cells [2]. In terms of prebiotic properties, β-glucans from *P. ostreatus* can stimulate the growth of beneficial gut bacteria such as *Lacticaseibacillus* and *Bifidobacterium*, helping to balance gut microbiota while promoting overall health [3]. Consequently, β-glucans have gained significant attention from the nutraceutical and pharmaceutical industries [4].

Among fungal sources, yeast and mushrooms are particularly notable for β-glucan production. Byproducts from brewing and baking industries using *Saccharomyces cerevisiae* are currently a major commercial source of β-glucans, primary structural components of the yeast cell wall. β-glucans from the yeast cell wall are composed mainly of β-1,3-D-glucan (≈85%) with β-1,6-D-glucan branches (≈15%) [5]. However, the multi-step extraction and purification processes required to isolate these compounds can be complex and costly, prompting a search for alternative sources and production methods [6]. While yeast-derived β-glucans are well-studied, mushrooms such as *P. ostreatus*, commonly known as the Oyster mushroom, may offer higher yields of β-glucans, especially when employing optimized extraction methods [7].

Mushrooms represent another excellent source of bioactive β-glucans. Species such as *Grifola frondosa*, *Lentinula edodes*, and *Pleurotus ostreatus* are known to contain significant amounts of β-glucans, ranging from 22 to 38% *w*/*w*, with *P. ostreatus* often exhibiting the highest levels [8,9]. *P. ostreatus* is one of the most widely cultivated edible mushrooms globally [10]. Such prominence stems from its rapid growth, ability to colonize diverse lignocellulosic substrates, and rich content of bioactive compounds, particularly β-glucans [11].

*P. ostreatus* is conventionally cultivated on low-value lignocellulosic substrates such as rice straw or sawdust [12]. This study explores a nutrient-rich alternative to enhance β-glucan production. Germinated Riceberry rice, rich in anthocyanins and GABA, offers a unique substrate that could improve both the yield and bioactivity of β-glucans [3]. This study, therefore, hypothesizes that the solid-state fermentation of *P. ostreatus* on germinated Riceberry rice will result in higher yields and purities of β-glucans compared to the fruiting body of the mushroom [13].

Furthermore, this study focuses on mycelial culture rather than the cultivation of fruiting bodies. Mycelial culture in controlled fermentation systems offers several advantages, including shorter cultivation times, scalability, and more consistent production and extraction of target compounds [13,14]. Solid-state fermentation (SSF) on rice-based media is a proven strategy for producing bioactive compounds, and mushroom mycelia, including *P. ostreatus*, have been reported as excellent sources of prebiotic compounds [14]. This study aims to optimize SSF conditions to maximize β-glucan yield from *P. ostreatus* grown on germinated Riceberry rice, while also assessing its prebiotic potential and anti-colorectal cancer activity.

## 2. Results

### 2.1. Optimal Fermentation Time for β-Glucan Production

**Figure 1:**
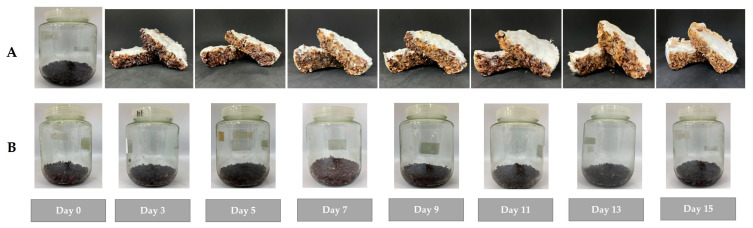
Visual comparison of solid-state fermentation over 15 days. (**A**) Germinated Riceberry rice fermented with *P. ostreatus* mycelium. (**B**) Unfermented germinated Riceberry rice (control).

After inoculating the mycelium culture on germinated Riceberry rice, the growth of fungal mycelium was observed from day 3 onward (Figure 1).

**Figure 2:**
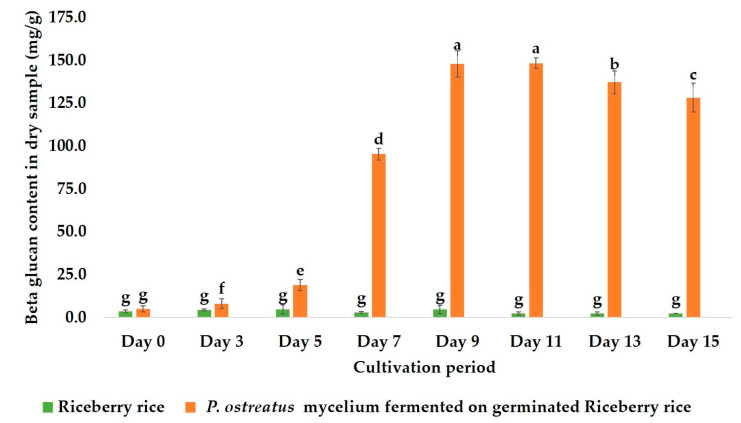
β-glucan content (mg/g dry weight) in *P. ostreatus*-fermented Riceberry rice from day 3 to day 15. The control bar represents unfermented rice. Values are mean ± standard deviation. Within each bar, values with different superscript letters (a–g) are significantly different (*p* < 0.05).

The β-glucan content in the fermented rice was quantified every two days. As shown in Figure 2, the β-glucan concentration increased dramatically during fermentation, reaching a maximum value of 148.04 ± 7.68 mg/g (dry weight) on days 9–11. This represents a more than 300-fold increase compared to the baseline level in the unfermented control rice (0.48 ± 0.10 mg/g). Extending the fermentation beyond this point resulted in a slight decline in β-glucan content. Therefore, a nine-day fermentation period was identified as the optimal point and used for all subsequent extractions, due to the shorter cultivation period than day 11.

### 2.2. Chemical and Physical Characterization of Extracted β-Glucan

#### 2.2.1. Extraction Yield and Purity

A yield of crude extract of 54.95 ± 0.63% *w*/*w* was obtained from the optimally fermented (day 9) substrate. Thermogravimetric analysis (TGA) was performed to assess the polysaccharide purity of the extract by comparing its thermal decomposition profile to a commercial β-(1,3-1,6) glucan standard. As detailed in Table 1, the purity of the crude β-glucan in this study was determined to be 79.98% *w*/*w*, slightly higher than the measured purity of the commercial standard (76.93% *w*/*w*). This technique was used to identify the total β-glucan content found in crude extract by measuring the weight change when heated under controlled conditions. The percentage of β-glucan was assessed from the remaining residue (68.59% *w*/*w*). After accounting for the percentage of β-glucan, the final crude extract containing a total of β-glucan was 37.69% *w*/*w*.

#### 2.2.2. Fourier-Transform Infrared (FTIR) Spectroscopy Analysis

**Figure 3:**
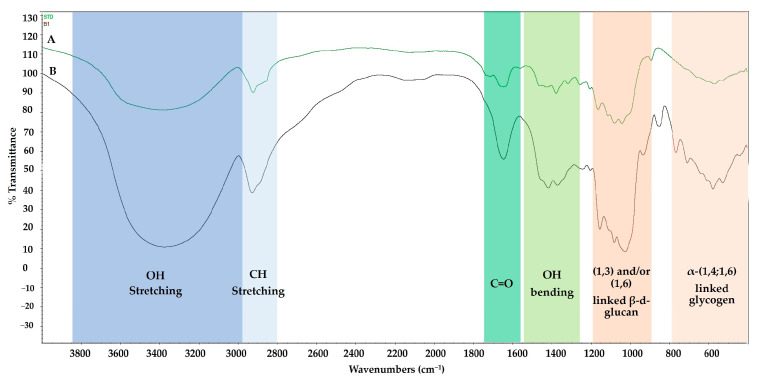
FTIR spectrum from 4000 to 400 cm^−1^ of standard β-glucan (1,3;1,6) (A) compared to crude β-glucan from mycelium on day 9 (B).

The molecular structure of the extracted β-glucan was analyzed by FTIR spectroscopy to identify characteristic functional groups and confirm its identity (Figure 3). The spectrum of the crude extract showed a strong resemblance to the β-glucan standard, confirming its polysaccharide nature. Key absorption bands included: A broad, strong peak at ~3298 cm^−1^, corresponding to the O-H stretching vibrations of the polysaccharide structure. Peaks in the 2880–2923 cm^−1^ range, indicative of C-H stretching vibrations. An absorption band of ~1640 cm^−1^ was likely due to the associated water or amide I band of residual proteins from the mycelia. A strong, complex absorption region between 1000 and 1200 cm^−1^, the “fingerprint” region for carbohydrates, confirmed the presence of C-O-C and C-OH groups in a pyranose ring structure. Crucially, the spectrum displayed a characteristic peak at ~891 cm^−1^, the definitive signature of β-glycosidic linkages, confirming that the extracted polysaccharide contained β-glucan. The spectral data strongly support the successful extraction of a (1,3-1,6)-β-D-glucan from the fermented Riceberry rice. However, the extract also contained α-glucan as indicated by a peak at ~593 cm^−1^.

### 2.3. Prebiotic Properties of Crude β-Glucan Extract

The prebiotic potential of the crude β-glucan was evaluated by its ability to selectively promote the growth of three probiotic bacterial strains (*Lacticaseibacillus rhamnosus*, *Bacillus coagulans*, and *Bifidobacterium longum*) over a pathogenic strain (*Escherichia coli*). This potential was quantified using two standard metrics: the Prebiotic Index (PI), which measures growth promotion relative to a control, and the Prebiotic Activity Score (PAS), which assesses probiotic selectivity over pathogens [15,16]. Lactic acid bacteria (LAB) are well-known probiotics that reach a log phase at around 8–12 h, depending on the species, with most LAB entering a stationary phase at around 24 h and then subsequently declining, suggesting that prebiotic potential can be determined at 24 h [16,17].

#### 2.3.1. Prebiotic Index (PI)

The PI values indicate how effectively a substrate supports probiotic growth compared to lactose control and no sugar supplement (negative control). Generally, a PI value less than or equal to 1 suggests low effectiveness or no significant stimulation compared to the control (lactose).

As shown in Table 2, the crude β-glucan extract demonstrates significant prebiotic activity. The extract was exceptionally effective at promoting the growth of *B. coagulans*, achieving a PI value of 115.70 ± 10.19. This was significantly higher (*p* < 0.05) than the values for the commercial β-glucan standard (102.49 ± 2.17) and widely used prebiotic inulin (90.53 ± 2.28).

For *L. rhamnosus*, the proposed extract also showed strong performance (PI = 6.36 ± 0.72), substantially outperforming the β-glucan standard (1.84 ± 0.15) and inulin (0.41 ± 0.09). The growth-promoting effect on *B. longum* was more modest but still positive (PI = 1.43 ± 0.02). In contrast, the unprocessed Riceberry rice and unextracted fermented mycelium showed negligible or inhibitory effects on probiotic growth.

#### 2.3.2. Prebiotic Activity Score (PAS)

The PAS was calculated to confirm that the growth promotion was selective (Table 3). A positive PAS value indicates that a substrate preferentially supports probiotic growth over pathogen growth. The crude β-glucan extract in this study exhibited positive PAS values for all three probiotic strains, confirming its selective prebiotic nature. The highest selectivity was observed for *B. coagulans* (PAS = 1.39 ± 0.06), a score comparable to that of inulin (1.41 ± 0.03). The extract also demonstrated powerful selectivity for *L. rhamnosus* (PAS = 0.56 ± 0.03), an effect significantly stronger than that of both inulin (0.14 ± 0.02) and the β-glucan standard (0.16 ± 0.03).

These results confirm that the crude β-glucan produced via SSF is a highly effective and selective prebiotic, with particularly potent activity toward *B. coagulans* and *L. rhamnosus.*

### 2.4. Anti-Colorectal Cancer Properties of Crude β-Glucan

#### 2.4.1. Effects of Crude β-Glucan on the Cell Viability of Colorectal Cancer Cell Lines (SW480) and Normal Colon Cell Lines (CCD841 CoN)

**Figure 4:**
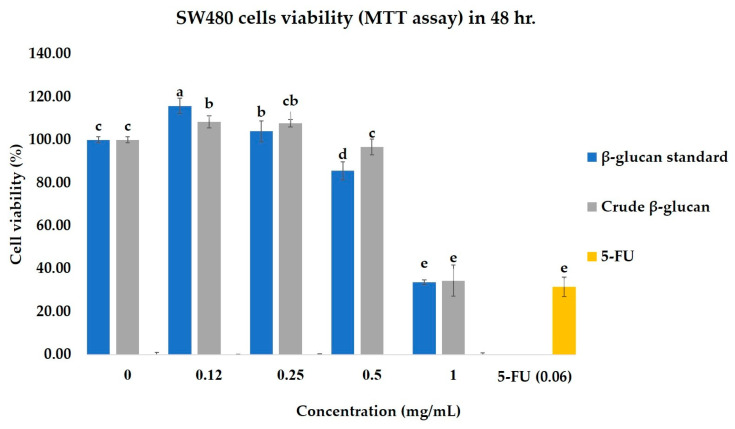
Viability of SW480 colon cancer cells after 48 h treatment. Within each bar, values with different superscript letters (a–e) are significantly different (*p* < 0.05). Vehicle control is 0 mg/mL.

**Figure 5:**
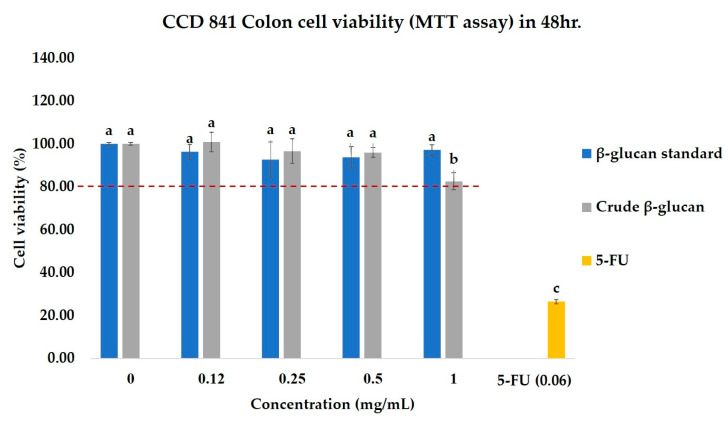
Viability of normal CCD841 CoN colon cells after 48 h treatment, demonstrating the low cytotoxicity of β-glucan extracts. Within each bar, values with different superscript letters (a–c) are significantly different (*p* < 0.05). Vehicle control is 0 mg/mL.

After 48 h of treatment, the crude β-glucan (1 mg/mL) significantly reduced the viability of SW480 cancer cells to 33.77 ± 2.42% (Figure 4). This potent cytotoxic effect was comparable to that of the commercial β-glucan standard (34.43 ± 7.20%) and the conventional chemotherapeutic drug, 5-fluorouracil (5-FU), at a concentration of 0.06 mg/mL. To assess selectivity, the same treatments were applied to the normal CCD841 CoN cell line (Figure 5). In stark contrast to their effect on cancer cells, both the crude β-glucan and standard β-glucan exhibited minimal toxicity, with cell viability remaining high at over 80%. This demonstrates a high degree of selectivity, especially when compared to 5-FU, which was highly toxic to the normal cells, reducing their viability to just 26.44%. These results indicate that the crude β-glucan possesses potent and selective anti-cancer activity, effectively killing cancer cells while sparing normal cells.

#### 2.4.2. Effects of Crude β-Glucan to Induce Apoptosis in Colon Cancer Cells (SW480) and Normal Colon Cells (CCD841 CoN)

**Figure 6:**
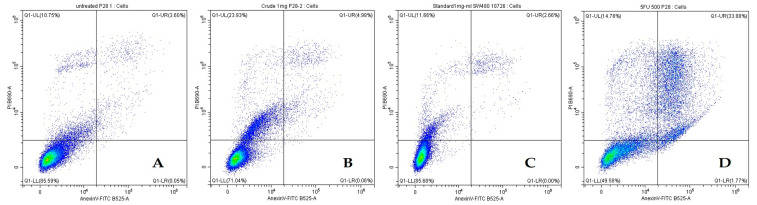
Flow cytometry analysis showing the minimal induction of apoptosis in SW480 colon cancer cells by β-glucan treatments compared to the high toxicity of 5-FU. (**A**) Untreated, (**B**) Crude β-glucan, (**C**) β-glucan standard, (**D**) 5-FU.

The effect of crude β-glucan on inducing cell death was investigated using a flow cytometer (Figure 6). The result in SW480 cells showed that 1 mg/mL of crude β-glucan induced greater cell death than the untreated control and 1 mg/mL of β-glucan standard, with total cell death percentages of 27.94 ± 0.93%, 14.26 ± 2.08%, and 13.77 ± 0.73%, respectively (Table 4). Unfortunately, the effect of crude β-glucan on inducing cell death was significantly lower than that of 5-FU, which induced 49.99 ± 1.54% of total cell death (Table 4).

**Figure 7:**
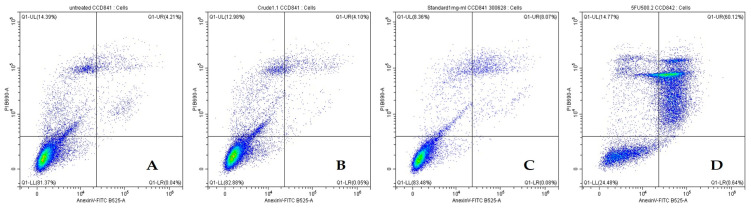
Flow cytometry analysis showing the minimal induction of apoptosis in normal CCD841 CoN cells by β-glucan treatments compared to the high toxicity of 5-FU. (**A**) Untreated, (**B**) Crude β-glucan, (**C**) β-glucan standard, (**D**) 5-FU.

To validate the safety of crude β-glucan, the effect of crude β-glucan in inducing total cell death was investigated in normal colon CCD841 CoN cells (Figure 7). The result showed that 1 mg/mL of crude β-glucan and β-glucan standard slightly induced cell death (15.95 ± 2.18 and 16.04 ± 0.56, respectively), consistent with the results from the cell viability assay (Figure 5). Notably, both crude β-glucan and β-glucan standard were less effective at inducing cell death than the untreated control (15.87 ± 0.51%) and 5-FU (73.90 ± 1.19%), as shown in Table 5.

## 3. Discussion

This study successfully demonstrates that SSF on a nutrient-rich substrate of germinated Riceberry rice is an effective strategy for producing bioactive β-glucans from *P. ostreatus* mycelium. Colored rice varieties are rich in proteins, fiber, and antioxidants that can nourish fungal growth [18]. By leveraging this enhanced substrate, the nine-day mycelial fermentation in this study achieved a β-glucan content of 148.04 mg/g, close to the β-glucan content of commercially available mushroom fruiting bodies (169.17 mg/g) measured by the same method. In comparison, research by Bualoi et al. (2021), which used sawdust mixed with rice straw, cultivated for 30 days to produce mushroom fruiting bodies (*P. ostreatus*), yielded only 55.17 mg per gram, less than one-third of the yield in this study [19] this could due to the direct quantification of (1→3, 1→6)-β-glucan in our study provides a more accurate measurement than the indirect Megazyme assay (K-YBGL) used in the prior study, which determines β-glucan by difference in total-glucan and alpha-glucan (α-glucan). However, the estimated β-glucan content may vary depending on different conditions (source, temperature, humidity, cultivation media), even for the same mushroom species. The process of cultivating *P. ostreatus* mycelium on germinated Riceberry rice is shorter in duration yet achieves comparable β-glucan amounts to mushroom fruiting bodies grown for longer periods. The subsequent extraction yielded a product with 79.98% purity, comparing favorably with the typical yields reported from various mushroom sources [9], underscoring the efficiency of the production pipeline in this study.

Structural analysis of crude β-glucan from powdered mycelium by FTIR confirmed the identity of the extracted product. The spectrum revealed characteristic absorption peaks for (1→3)-β-D-glucan (1072–1078 cm^−1^) and (1→6)-β-D-glucan linkages (993–994 cm^−1^), which is consistent with the known structure of polysaccharides from *P. ostreatus* [20,21]. The prominent peak at ~891 cm^−1^ is the definitive signature of β-glycosidic bonds. The potential presence of minor residual α-glucans (e.g., starch) from the Riceberry substrate is consistent with the extract’s ~83% purity and may account for other minor signals [22].

**Figure 8:**
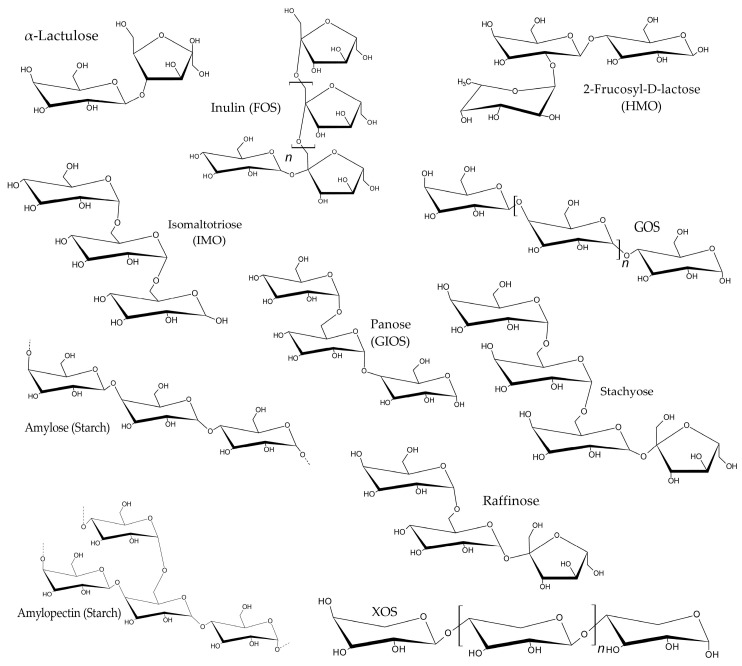
Structure of prebiotics in each group [25].

**Figure 9:**
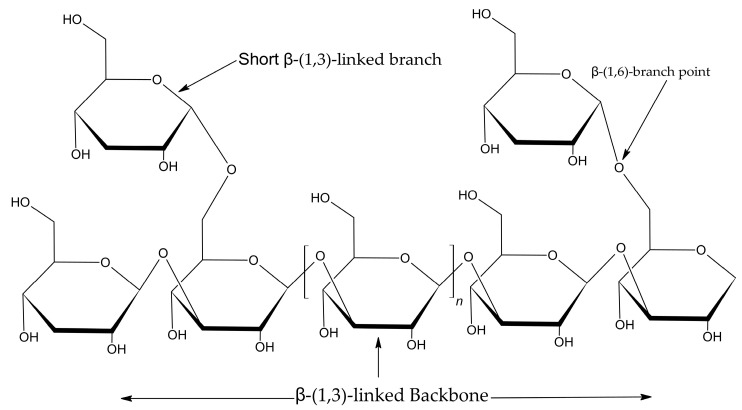
Structure of β-glucan [43].

Previous reports have shown that β-glucan possesses prebiotic and anti-colon cancer properties [23]. Prebiotics such as β-glucan, inulin, and fructo-oligosaccharides (FOS) are essential for a healthy gut by maintaining the homeostasis of gut microbiota and inducing the synthesis of healthy short-chain fatty acids (SCFAs) [17,24]. As shown in Figure 8, each prebiotic promotes growth on specific probiotic bacteria due to its distinct enzymes [25]. Figueroa-González (2019) reported that commercial prebiotics significantly promoted the growth of *L. rhamnosus* over other probiotic strains, with a PI value of 7.22 [16]. Moreover, Huebner et al. (2007) reported the prebiotic properties of commercial prebiotics on two groups of bacteria, namely *Lacticaseibacillus* and *Bifidobacterium* [26]. Therefore, *L. rhamnosus*, *B. coagulans*, and *B. longum* were employed as models to determine the prebiotic properties of the crude β-glucan, with inulin used as the standard prebiotic. The results indicated that the crude β-glucan from mycelium exhibited prebiotic properties consistent with a previous report from a commercial prebiotic, in which crude β-glucan promoted the growth of *L. rhamnosus* better than inulin, with PI and PAS values of 6.36 ± 0.72 and 0.56 ± 0.03, respectively [16]. In addition, the crude β-glucan also effectively promoted the growth of *B. coagulans*, with PI and PAS values of 115.70 ± 10.19 and 1.39 ± 0.06, respectively. In contrast, inulin was more effective at promoting the growth of *B. longum* than the crude β-glucan, aligning with data from Optibacprobiotics. Prebiotics, such as FOS and inulin, effectively stimulate the growth of *Bifidobacterium* strains since their enzymes can digest FOS and inulin. *B. longum* produces enzymes that specifically digest inulin and related FOS. This enzymatic compatibility allows better fermentation and metabolite production, supporting probiotic growth. β-glucans have a more complex structure than FOS, which limits their utilization by *B. longum*, making the growth difference biologically relevant and expected [27]. Since *B. coagulans* has some enzymes (e.g., beta-glucanase) that can break down β-glucan into simpler sugars, *B. coagulans* possibly uses β-glucan as an energy source to promote its growth. The presence of β-glucan increases the growth and activity of *B. coagulans* due to a strong interaction between β-glucan and the cell wall of *B. coagulans* [28]. Unlike *B. coagulans*, *Bifidobacteria* and *Lactobacillus* cannot effectively metabolize β-glucan due to different metabolic pathways. Since β-glucan has a complex polysaccharide structure with intricate branching patterns (Figure 9), its complexity may hinder certain probiotic strains from utilizing β-glucan due to a lack of necessary enzymes for digestion. Aulitto et al. (2021) reported that the key enzyme used by *B. coagulans* to digest β-glucan is β-glucanase [29], whereas there are no reports of *L. rhamnosus* producing enzymes to digest β-glucan itself. However, some glycosyl hydrolases (such as glucosidase enzymes) have been reported to be the enzymes used by LAB to degrade β-glucan [30]. These prebiotic findings demonstrate that crude β-glucan from mycelium acts as a selective prebiotic, effectively promoting the growth of beneficial probiotics such as *L. rhamnosus* and *B. coagulans*. This selective stimulation is likely due to the presence of specific enzymes like β-glucanase in *B. coagulans* and potential glucosidase activity in LAB, supporting targeted modulation of gut microbiota and overall gut health. However, the crude β-glucans extract was not purified, so the extract contained both α- and β-glucans. According to Synytsya et al. (2014) [22] and Brust et al. (2020) [31], the structure of α-glucans is connected by α bonds, resulting in a flexible helical structure, which is more suitable for compact energy storage in the cells rather than acting as a prebiotic since the structure is not resistant to digestion by enzymes in the body. On the other hand, β-glucans have a rigid and complex structure, making them resistant to digestion by human enzymes and inducing prebiotic properties [22,31]. Moreover, some data suggest that when α-glucans undergo hydrolysis, their prebiotic efficacy improves due to increased resistance to digestion. This aligns with the research by Tan et al. (2022), who reported that hydrolyzed α-glucans better promote the growth of probiotics than prior to hydrolysis [32]. Therefore, the prebiotic effects that promote the growth of probiotic strains in this study are likely due to the β-glucans rather than the α-glucans in the extracted crude.

According to the results in normal intestinal CCD841 CoN cells, β-glucans from *P. ostreatus* revealed low cytotoxicity, consistent with a previous report on β-glucans from *F. betulina* [33], suggesting that crude β-glucans from *P. ostreatus* are possibly safe and edible. Liu (2023) and Shamekhi et al. (2020) demonstrated that mushroom extracts reduce the viability of SW480 colon cancer cells [34,35]. The results of this present study show that 1 mg/mL of crude β-glucan is able to reduce the viability of SW480 cells by up to 36% after 48 h of treatment, consistent with a previous report that a crude β-glucan concentration of 0.4 mg/mL could inhibit SW480 cells by up to 40% [35]. The effect of crude β-glucan against SW480 cells was confirmed using flow cytometry. Notably, the finding that 1 mg/mL crude β-glucan induces cell death (27.94 ± 0.93%) correlates with the previous cell viability result. In addition, (1,3-1,6)-β-glucans from *Fomitopsis betulina* have been reported to decrease the viability of the Caco-2 colon cancer cell line [36]. Zade et al. (2025) reported that β-glucan from *P. ostreatus* can inhibit the proliferation of SW480 cells, but with no clear evidence of the mechanism of cell death [37]. According to the flow cytometry result, the mechanism of cell death was not classified as apoptosis. Lactosylceramide (LacCer) is a glycosphingolipid that is expressed on the membrane of neutrophils and endothelial cells. (1,3-1,6)-β-glucans have been reported to interact with the LacCer to trigger neutrophile chemotaxis against *Candida albicans* [38]. The interaction of LacCer with β-glucans also triggers oxidative stress or oxidative burst through a MIP-2/TNF-α/NF-κB/PKC cascade that further intricately modulates several programs of cell death, including apoptosis, necrosis, ferroptosis, pyroptosis, etc. [39]. The overexpression of lactosylceramide synthase β-1,4-GalT-V has been reported in human colorectal cancer, suggesting that (1,3-1,6)-β-glucans possibly kill colon cancer cells through several cell death mechanisms [40]. Therefore, further investigation is required to validate the mechanism of cell death promoted by (1,3-1,6)-β-glucans from *P. ostreatus*.

According to the results, crude β-glucan has a stronger inhibitory effect against colon cancer cells compared to the β-glucan standard from yeast. Although β-glucans from mushrooms and yeast are (1,3-1,6)-β-glucans, the structural complexity and branching patterns of β-glucans from mushrooms differ from those of yeast. Varnosfaderani et al. (2024) reported that these structural complexities and branching patterns influence the effectiveness of β-glucan to stimulate immune cells such as macrophages, NK cells, and dendritic cells [39]. The findings of Han et al. (2020) [41] reveal that the β-(1,3)-linked backbone is essential for recognition by immune receptors such as Dectin-1. A minimal chain length of approximately seven glucose units is required for effective receptor binding. Side chains at the β-(1,6) position add branched structures that modulate binding affinity and biological activity [41]. Another reason for the extract having better inhibitory effects on colon cancer than the β-glucan standard from yeast is that the extract is not a purified substance, and structural analysis using FTIR, as shown in Figure 3, shows the presence of both β- and α-glucans. Lavi et al. (2006) report that polysaccharide extracts (α-glucan-rich) from *Pleurotus ostreatus* induce apoptosis in colon cancer cells (HT-29) by increasing the Bax/Bcl-2 ratio, triggering mitochondrial cytochrome c release, and activating the caspase-9/caspase-3 pathway [42]. Therefore, the enhanced inhibition of colon cancer cells by crude β-glucan in comparison with the β-glucan standard from yeast may be due to the synergistic action of both β- and α-glucans in the extract. Interestingly, the results from the cell viability assay and flow cytometry showed low toxicity in normal intestinal CCD841 CoN cells, indicating the specificity of crude β-glucan to inhibit colon cancer cells.

## 4. Materials and Methods

### 4.1. Materials

The *Pleurotus ostreatus* strain was sourced from a local mushroom farm in Chiang Rai, Thailand. Riceberry rice (*Oryza sativa* L.) was purchased from a Chiang Rai farmers’ cooperative in the same region. Commercial standards, including β-(1,3-1,6)-glucan (from yeast) and inulin, were purchased from Sigma Aldrich (St. Louis, MO, USA). All microbial culture media, including Potato Dextrose Agar (PDA), Potato Dextrose Broth (PDB), and de Man, Rogosa, and Sharpe (MRS) agar, were procured from Hi-Media Laboratories (Mumbai, India). All other chemicals and reagents were of analytical grade.

### 4.2. Fungal Culture and Solid-State Fermentation

#### 4.2.1. Inoculum and Riceberry Rice Media Preparation

The *P. ostreatus* mycelium was maintained on PDA plates and incubated at 30 °C in 60% humidity for 10 days. For liquid inoculum preparation, 15 mycelial plugs (5 mm in diameter) from the PDA culture were transferred to a 500 mL Erlenmeyer flask containing 300 mL of PDB and incubated at 30 °C for seven days in an orbital shaker at 125 rpm.

The Riceberry rice substrate was prepared using 25 g of grains by soaking them in 25 mL reverse osmosis (RO) water (1:1, *w*/*v*, pH 6–7) in a 300 mL bottle for 16 h to induce germination. The soaked, germinated rice was then sterilized by autoclaving at 121 °C for 15 min.

#### 4.2.2. Solid-State Fermentation (SSF)

The sterilized, germinated Riceberry rice was inoculated with the liquid mycelial culture at a ratio of 10% (*v*/*w*). At the same time, the germinated Riceberry rice control was not inoculated with mycelium culture. Both substrates were incubated at 30 °C in 60% humidity for 15 days. To determine the optimal fermentation time for β-glucan production, samples were collected from three bottles at a time (50 g of wet sample/bottle) on days 3, 5, 7, 9, 11, 13, and 15. The collected samples were dried in a hot air oven at 60 °C for 48 h and then milled into a fine powder. The reproducibility of fermentation was repeated more than three times.

### 4.3. β-Glucan Extraction

A microwave-assisted extraction (MAE) method was used to isolate crude β-glucan. 10 g of the dried fermented powder was suspended in a 500 mL bottle containing 200 mL RO water at a ratio of 1:20 (*w*/*v*). The suspension was heated in a microwave oven at 900 W for 5 min. After heating, the mixture was centrifuged (6000× *g*, 10 min) to separate the supernatant. The remaining pellets were subjected to a second round of extraction under the same conditions. The supernatants from both extractions were pooled and lyophilized (freeze-dried) at −80 °C for 0.01 mbar to obtain the crude β-glucan powder.

### 4.4. Analytical Characterization

#### 4.4.1. β-Glucan Quantification

The β-glucan content was quantified using the Enzymatic Yeast β-Glucan Assay Kit (K-EBHLG, Megazyme, Wicklow, Ireland) following the manufacturer’s protocol. Briefly, the milled sample and standard β-glucan (20 ± 0.1 mg) were placed into a glass test tube (16 × 100 mm). Samples were solubilized in 0.4 mL of 2 M KOH (Qrec, Auckland, New Zealand) and neutralized using 1.6 mL of a 1.2 M sodium acetate buffer. The β-glucan polymers were then enzymatically hydrolyzed to glucose using a mixture of high-purity *exo*-1,3-β-glucanase and β-glucosidase. Incubate at 40 °C in a water bath (Memmert WNE-22, Schwabach, Germany) for 16 h. The total glucose released was measured spectrophotometrically (Thermo, Waltham, MA, USA, Evolution 200 series) at 510 nm using a glucose oxidase/peroxidase (GOPOD) reagent. The β-glucan percentage was calculated using the formula provided by the manufacturer (Equation (1)). The experimental procedures were carried out in triplicate (*n* = 3)

(1) $$\beta \text{-}\mathrm{Glucan}\;(\%\;w/w)=\Delta A\;\times \;F\;\times \;\frac{12.04}{0.1}\;\times \;\frac{100}{W}\;\times \;\frac{1}{1000}\;\times \;\frac{162}{180}$$

ΔA = Absorbance read against reagent blank.

F = Conversion from absorbance to µg (150 µg of D-glucose standard divided by the GOPOD absorbance of this 150 µg).

W = Weight of sample analyzed in mg.

#### 4.4.2. Thermogravimetric Analysis (TGA)

The thermal stability and purity of the extract were assessed by TGA using a TGA analyzer (PerkinElmer, Waltham, MA, USA), following the method used by Suraiya et al. (2024) [44]. Approximately 10 mg of the dried sample was heated in an alumina crucible from 50 °C to 800 °C at a rate of 10 °C/min under a continuous nitrogen flow of 25 mL/min. The resulting thermogravimetric curve, displaying the percentage weight loss versus temperature, was used to evaluate the thermal stability of the polysaccharides [44].

#### 4.4.3. Fourier-Transform Infrared (FTIR) Spectroscopy

FTIR is a powerful analytical technique used to identify and characterize materials based on their infrared absorption patterns. The crude β-glucan extract was analyzed using FTIR spectroscopy within the frequency range of 4000–400 cm^−^**^1^**, employing a Spectrum X instrument (Perkin Elmer, USA) based on the method employed by Suraiya et al. (2024) [44]. The dried β-glucan was individually placed on the attenuated total reflection (ATR) crystal, with pressure applied to eliminate air from the powder particles. The final spectrum was obtained by averaging 128 scans at four different resolutions [44].

### 4.5. Prebiotic Activity Assays

#### 4.5.1. Probiotic Growth Stimulation

The prebiotic potential was tested using three probiotic strains: *Lacticaseibacillus rhamnosus*, *Bacillus coagulans*, and *Bifidobacterium longum*. *Escherichia coli* served as the pathogenic control. Strains were grown in a basal medium (0.1% peptone and 0.1% yeast extract) supplemented with 1% (*w*/*v*) of the test substrate in a 250 mL Erlenmeyer flask containing 100 mL of media (crude β-glucan, inulin, etc.). The microbial strains were inoculated at a concentration of 1 mL (1% *v*/*v*) into prebiotic media and incubated at 37 °C at 200 rpm for 48 h. The culture medium was collected for 5 mL every 4 h to determine the number of colony-forming units (CFU/mL) by counting colonies on MRS plates for the probiotic strains and nutrient agar plates for *E. coli*. The plates were incubated at 37 °C for 24–48 h [16]. The experimental procedures were carried out in triplicate (*n* = 3).

#### 4.5.2. Prebiotic Index (PI) and Prebiotic Activity Score (PAS)

The PI and PAS were calculated to quantify prebiotic efficacy, as described by Figueroa-González et al. [16]. The PI and PAS were used to determine the potential activity of prebiotics promoting the growth of probiotics. The PI value was determined from Equation (2). A PI value close to 1 suggests low effectiveness of the evaluated carbohydrate, whereas a value higher than 1 indicates a positive impact on probiotic growth. PAS values were determined from Equation (3). When compared to lactose, a high PAS implies that probiotic bacteria can survive well. When grown on prebiotics rather than lactose, *E. coli* should develop at a slower pace. Therefore, the PAS may be calculated using Equation (3) in connection with a given probiotic strain. The growth value of *E. coli* in each supplement is only used to calculate the PAS value, which will then be used to compare the prebiotic activity of each probiotic strain to determine its ability to survive in a competitive environment.

(2) $$\mathrm{PI}=\frac{\mathrm{CFU}\;\mathrm{of}\;\mathrm{probiotic}\;\mathrm{in}\;\mathrm{prebiotic}\;\mathrm{carbohydrate}}{\mathrm{CFU}\;\mathrm{of}\;\mathrm{probiotic}\;\mathrm{in}\;\mathrm{control}\;\mathrm{carbohydrate}}$$

(3) $$\mathrm{PAS}=\frac{(\mathrm{LogP}24\;\text{-}\;\mathrm{LogP}0)\;\mathrm{prebiotic}}{(\mathrm{LogP}24\;\text{-}\;\mathrm{LogP}0)\;\mathrm{lactose}}-\frac{(\mathrm{LogE}24\;\text{-}\;\mathrm{LogE}0)\;\mathrm{prebiotic}}{(\mathrm{LogE}24\;\text{-}\;\mathrm{LogE}0)\;\mathrm{lactose}}$$

where PAS is a prebiotic activity score, LogP is the log of growth (CFU/mL) of the probiotic bacteria at 24 h (P24) and 0 h (P0) of culture on prebiotic and lactose, LogE is the log of growth (CFU/mL) of *E. coli* at 24 h (E24) and 0 h (E0) of culture on prebiotic and lactose [16].

### 4.6. Anti-Cancer Activities

#### 4.6.1. Cell Lines and Culture Conditions

The human colon adenocarcinoma cell line SW480 and the normal human colon cell line CCD841 CoN were obtained from the American Type Culture Collection (ATCC, Manassas, VA, USA). Both cell lines were maintained in Dulbecco’s Modified Eagle’s Medium (DMEM, Cytiva HyClone, Logan, UT, USA) supplemented with 10% Fetal Bovine Serum (FBS, Cytiva HyClone, USA) and 1% penicillin/streptomycin solution (Cytiva HyClone, USA), incubated at 37 °C in a humidified atmosphere with 5% CO_2_ [45].

#### 4.6.2. Cell Viability (MTT) Assay

Cell viability was assessed using the 3-(4,5-dimethylthiazol-2-yl)-2,5-diphenyltetrazolium bromide (MTT, Sigma Aldrich, Saint Louis, MO, USA) assay, adapted from Liu et al. (2023) [35]. Cells were seeded in 96-well plates (5 × 10^3^ cells/well) and allowed to adhere for 12 h. The medium was then replaced with fresh medium containing various concentrations of the test samples (0.125 to 1 mg/mL). After a 48 h incubation, 0.5 mg/mL of MTT reagent (10 µL) was added to each well and incubated for 4 h. The formazan crystals were dissolved in 100 µL of DMSO, and the absorbance was measured at 540 nm. Using a microplate reader (PerkinElmer Envision, USA). The percentage of cell viability was calculated relative to untreated control cells (Equation (4)). Colon cancer cells were used to test the cytotoxic effects on cancer cells, while normal colon cell lines served as indicators to determine whether the extract was toxic to normal cells.

(4) $$\%\;\mathrm{Viability}=\frac{\mathrm{absorbance}\;\mathrm{of}\;\mathrm{control}\;\text{-}\;\mathrm{absorbance}\;\mathrm{of}\;\mathrm{tested}\;\mathrm{sample}}{\mathrm{absorbance}\;\mathrm{of}\;\mathrm{control}\;}\times \;100$$

#### 4.6.3. Apoptosis Assay by Flow Cytometry

Cell apoptosis was quantified using an Annexin V-FITC/Propidium Iodide Apoptosis Detection Kit (E-CK-A211) according to the manufacturer’s protocol and adapted from Liu et al. (2023) [35]. Cells were seeded in 6-well plates (2.5 × 10^5^ cells/well), treated with various concentrations of the sample for 48 h, then harvested. The cell pellets were resuspended in a binding buffer and stained with Annexin V-FITC and PI for 15 min in the dark. The stained cells were analyzed immediately on a flow cytometer (Beckman Coulter, CytoFLEX SRT, Brea, CA, USA), counting 2 × 10^4^ events per measurement.

### 4.7. Statistical Analysis

Unless otherwise specified, all experimental procedures were carried out independently in triplicate (*n* = 3). For all cell culture experiments, five replicates (*n* = 5) were used, with the results expressed as mean ± standard deviation (SD). Statistical analyses were performed using IBM SPSS Statistics v23. Data values were normally distributed to perform statistical analysis. Data were analyzed by one-way analysis of variance (ANOVA), and means compared using Duncan’s multiple range test for post hoc analysis. Differences were considered statistically significant at *p* < 0.05.

## 5. Conclusions

This study details a new, scalable SSF process using *Pleurotus ostreatus* on germinated Riceberry rice, establishing an efficient nine-day method for producing high-purity (1→3, 1→6)-β-glucan. The developed process yielded a crude product with 54.95% β-glucan, which was further confirmed to have a purity of 79.98% through comprehensive physicochemical analyses (FTIR, TGA). The findings highlight significant translational value, as the SSF process is well-suited for industrial scale-up for β-glucan production from mushrooms due to its use of low-cost substrates and established fermentation technology. Functionally, this novel fermented product demonstrated significant biological activity. In vitro, it exhibited potent prebiotic properties, stimulating the growth of *Lactobacillus rhamnosus* and *Bacillus coagulans* at levels comparable to commercial inulin. Furthermore, it displayed promising anti-cancer effects, effectively inhibiting the proliferation of SW480 colon cancer cells at a non-toxic concentration (1 mg/mL). Finally, although the current study demonstrates promising in vitro prebiotic and anti-cancer activities of the fermented β-glucan product, there are limitations in extrapolating these results directly to in vivo contexts. Potential metabolic interferences in a living organism could modulate or alter the prebiotic efficacy and anti-cancer mechanisms observed in vitro. Therefore, future research should include in vivo validation to confirm these biological effects, evaluate bioavailability, and investigate possible postbiotic influences. Addressing these aspects is crucial for the successful application of this β-glucan in functional foods and nutraceutical products.

## 6. Patents

The upscaling β-glucan method from *P. ostreatus* was part of the Thailand patent registration number 2503003680.

## Figures and Tables

**Table 1:** TGA-based composition and purity analysis of crude β-glucan extract compared to a commercial standard.

Sample	Moisture (% *w*/*w*)	β-Glucan (% *w*/*w*)	Residue (% *w*/*w*)	Purity (%) *	Total of β-Glucan (% *w*/*w*)
Crude β-glucan	14.24	68.59	17.17	79.98	37.69
β-glucan standard	8.79	70.17	21.07	76.93	-

* Purity calculated on a dry-weight basis as [%β-glucan/(100%Moisture)] × 100.

**Table 2:** Prebiotic Index (PI) of various substrates on the growth of three probiotic strains.

Sample	Prebiotic Index		
	*L. rhamnosus*	*B. coagulans*	*B. longum*
No sugar supplement	0.37 ± 0.06 ^c^	0.45 ± 0.07 ^d^	1.48 ± 0.07 ^c^
Inulin	0.41 ± 0.09 ^c^	90.53 ± 2.28 ^c^	6.23 ± 0.32 ^b^
β-glucan standard	1.84 ± 0.15 ^b^	102.49 ± 2.17 ^b^	7.84 ± 0.19 ^a^
Riceberry rice	0.04 ± 0.00 ^c^	5.23 ± 0.36 ^d^	0.43 ± 0.01 ^d^
Mycelium	0.02 ± 0.01 ^c^	0.17 ± 0.14 ^d^	0.85 ± 0.59 ^d^
Crude β-glucan	6.36 ± 0.72 ^a^	115.70 ± 10.19 ^a^	1.43 ± 0.02 ^c^

Note: Data are presented as mean ± standard deviation (*n* = 3). Within each column, values with different superscript letters (a–d) are significantly different (*p* < 0.05).

**Table 3:** Prebiotic activity score of various substrates on the growth of three probiotic strains.

Sample	Prebiotic Activity Score (PAS)		
	*L. rhamnosus*	*B. coagulans*	*B. longum*
No sugar supplement	0.09 ± 0.04 ^b^	0.06 ± 0.07 ^d^	0.48 ± 0.10 ^c^
Inulin	0.14 ± 0.02 ^b^	1.41 ± 0.03 ^a^	1.22 ± 0.22 ^a^
β-glucan standard	0.16 ± 0.03 ^b^	1.13 ± 0.06 ^b^	0.91 ± 0.12 ^b^
Riceberry rice	−0.09 ± 0.02 ^c^	0.87 ± 0.05 ^c^	−0.12 ± 0.06 ^e^
Mycelium	−0.54 ± 0.07 ^d^	−0.15 ± 0.08 ^e^	0.29 ± 0.08 ^cd^
Crude β-glucan	0.56 ± 0.03 ^a^	1.39 ± 0.06 ^a^	0.21 ± 0.10 ^d^

Note: Data are presented as mean ± standard deviation (*n* = 3). Within each column, values with different superscript letters (a–e) are significantly different (*p* < 0.05).

**Table 4:** Quantification of apoptosis in SW480 cancer cells after 48 h treatment.

Treatments	Live Cell	Early Apoptosis Cell	Late Apoptosis Cell	Total Cell Death
Untreated	86.20 ± 0.71 ^a^	0.04 ± 0.01 ^b^	3.75 ± 0.22 ^b^	13.77 ± 0.73 ^c^
Crude β-glucan 1 mg/mL	71.80 ± 0.66 ^b^	0.07 ± 0.02 ^b^	4.93 ± 0.05 ^b^	27.94 ± 0.93 ^b^
β-glucan standard 1 mg/mL	85.58 ± 0.71 ^a^	0.01 ± 0.01 ^b^	2.76 ± 0.10 ^b^	14.26 ± 2.08 ^c^
5-FU 0.06 mg/mL	49.73 ± 1.07 ^c^	1.92 ± 0.39 ^a^	34.77 ± 2.42 ^a^	49.99 ± 1.54 ^a^

Note: Data are presented as mean ± standard deviation (*n* = 3). Within each column, values with different superscript letters (a–c) are significantly different (*p* < 0.05).

**Table 5:** Percentage of apoptosis in CCD841 CoN cancer cells using Flow Cytometry.

Treatments	Live Cell	Early Apoptosis Cell	Late Apoptosis Cell	Total Cell Death
Untreated	81.30 ± 0.61 ^b^	0.07 ± 0.04 ^b^	4.89 ± 1.21 ^c^	18.70 ± 0.62 ^b^
Crude β-glucan 1 mg/mL	83.85 ± 1.81 ^a^	0.08 ± 0.05 ^b^	4.83 ± 1.49 ^c^	15.95 ± 2.18 ^c^
β-glucan standard 1 mg/mL	83.68 ± 0.76 ^a^	0.08 ± 0.02 ^b^	7.44 ± 1.34 ^b^	16.04 ± 0.56 ^c^
5FU 0.06 mg/mL	25.44 ± 1.23 ^c^	0.66 ± 0.08 ^a^	60.54 ± 0.47 ^a^	74.56 ± 1.23 ^a^

Note: Data are presented as mean ± standard deviation (*n* = 3). Within each column, values with different superscript letters (a–c) are significantly different (*p* < 0.05).

## Data Availability

The data presented in this study are available on request from the corresponding author.
